# Comparative effectiveness of initial systemic versus intratympanic corticosteroid therapy for sudden sensorineural hearing loss: a retrospective cohort study with doubly robust analysis

**DOI:** 10.3389/fneur.2026.1752868

**Published:** 2026-04-15

**Authors:** Li Guo, Yang Li, Junli Wang, Ying Gao, Yuqi Feng, Baojun Wu, Xiaoyong Ren

**Affiliations:** 1 Department of Otolaryngology, The Second Affiliated Hospital of Xi’an Jiaotong University, Xi’an, China; 2 Shaanxi Provincial Key Laboratory for Precision Diagnosis and Treatment of Otorhinolaryngology, Xi’an, China

**Keywords:** augmented IPTW, causal inference, corticosteroids, intratympanic injection, inverse probability weighting, propensity score, sudden sensorineural hearing loss

## Abstract

**Background:**

The optimal initial corticosteroid strategy for idiopathic sudden sensorineural hearing loss (SSNHL) remains uncertain, and randomized trials may not capture real-world variability. Observational comparisons are vulnerable to confounding, particularly when post-treatment variables are inappropriately adjusted. We evaluated the real-world comparative effectiveness of initial systemic versus intratympanic corticosteroids using a prespecified causal inference framework.

**Methods:**

We conducted a retrospective cohort study (2012–2019) of adults with idiopathic SSNHL and complete baseline and discharge audiometry. Patients receiving combined systemic–intratympanic therapy or missing exposure data were excluded. The exposure was the initial corticosteroid route (systemic oral/IV vs. intratympanic). Propensity scores were estimated from baseline covariates including age, sex, symptom profile, comorbidities, baseline PTA, and time from symptom onset to treatment initiation. Primary estimation used augmented inverse probability of treatment weighting (AIPW) to retain all eligible patients; 1:1 propensity-score matching (caliper 0.2 SD of logit PS) with regression adjustment addressed residual imbalance as a sensitivity analysis. Outcomes were PTA gain (primary), complete recovery (≥30 dB gain), and effective improvement (≥15 dB gain).

**Results:**

Among 284 adults (systemic = 240; intratympanic = 44), crude mean PTA gain was 13.8 vs. 3.8 dB (difference 10.0 dB). In AIPW analysis, systemic therapy was associated with greater PTA gain (adjusted mean difference 10.1 dB; 95% bootstrap CI 1.8–19.5) and a higher probability of complete recovery (adjusted risk difference 16.9%; 95% CI 9.4–23.4). The risk difference for effective improvement was 11.4% (95% CI − 15.3 to 30.9). Findings were directionally consistent in IPTW, overlap-weighted, and matched analyses.

**Conclusion:**

In this single-center real-world cohort, initial systemic corticosteroids were associated with greater short-term hearing improvement and a higher likelihood of complete recovery compared with intratympanic therapy. Weighting-based doubly robust estimators improved precision and generalizability, while matched analyses yielded consistent direction but wider uncertainty.

## Introduction

Sudden sensorineural hearing loss is an otologic emergency characterized by acute (≤72 h) loss of hearing in one or both ears. Corticosteroids are the mainstay of treatment and can be delivered systemically, intratympanically, or via combined routes. Although randomized trials and meta-analyses have compared these approaches, strict inclusion criteria, protocolised dosing and selective use of salvage therapy may limit generalizability. Real-world evidence is therefore needed to inform the effectiveness of initial steroid strategies across the spectrum of patient presentations.

Prior observational studies of SSNHL have used propensity score methods to emulate randomised comparisons, but many reports provide limited information on propensity model specification, balance diagnostics, or handling of post-treatment variables (1). Treatment intensity measures such as total dose and duration are sometimes included in propensity score models despite being determined after the initial treatment route is chosen; such post-treatment adjustment can induce bias and violate temporal ordering assumptions required for causal inference (2, 3). Randomised trials comparing oral and intratympanic corticosteroids in selected idiopathic SSNHL populations have suggested no large differences in hearing outcomes (4). Systematic reviews and meta-analyses have likewise reported no clear superiority of either route as primary therapy, although the certainty of evidence is often low and heterogeneity is substantial (5–8), leaving ongoing clinical equipoise. The 2019 AAO-HNSF guideline update recommends offering corticosteroids within 2 weeks of symptom onset and endorses intratympanic steroid therapy as salvage treatment for incomplete recovery within 2–6 weeks (9). Additional clinical studies and guideline reports have addressed intratympanic steroid dose effects, oral versus intratympanic regimens, age-related differences, and prognostic factors in SSNHL (18–23). Against this background, we evaluated the real-world comparative effectiveness of initial systemic versus intratympanic corticosteroids using a prespecified causal inference framework. We (i) restricted propensity score estimation to baseline covariates (including time to treatment), (ii) assessed balance using absolute standardised mean differences and Love plots, and (iii) applied doubly robust estimators that combine propensity score adjustment with outcome regression—augmented inverse probability of treatment weighting (AIPW) as the primary analysis and propensity-score matching with regression adjustment as a sensitivity analysis (10, 11).

## Methods

### Study design and setting

This single-centre retrospective cohort study was conducted at a tertiary otology referral centre in Xi’an, China. Electronic health records were searched to identify all adult patients (≥18 years) diagnosed with idiopathic SSNHL between 1 January 2012 and 31 December 2019. The study protocol adhered to the Strengthening the Reporting of Observational Studies in Epidemiology (STROBE) guidelines (1).

### Participants

Idiopathic SSNHL was defined as a sensorineural hearing loss of ≥30 dB affecting at least three consecutive frequencies occurring within 72 h, with no identifiable cause after standard clinical evaluation, consistent with AAO-HNSF guideline criteria (9).

Inclusion criteria were: (1) adults (≥18 years) diagnosed with idiopathic SSNHL; (2) received initial corticosteroid therapy delivered via a single route—systemic (oral or intravenous) or intratympanic injection—during the index hospitalization; and (3) complete pure-tone audiometry at admission and discharge (or the last in-hospital audiogram). Exclusion criteria were: (1) secondary causes of SSNHL identified from clinical evaluation and investigations; (2) missing exposure data; (3) combined systemic plus intratympanic therapy during the index treatment course; and (4) incomplete baseline or discharge audiometry.

### Variables and data sources

Baseline data were extracted from electronic medical records and included demographics (age, sex), symptom profile (tinnitus, ear fullness, vertigo), comorbidities (hypertension, coronary heart disease, diabetes), time from symptom onset to treatment initiation, and audiometry. The baseline pure-tone average (PTA) was calculated as the mean of 500, 1,000, 2000 and 4,000 Hz thresholds measured at admission; discharge PTA was calculated from the corresponding frequencies on the last in-hospital audiogram. Exposure was the route of initial steroid administration (systemic vs. intratympanic). Treatment intensity (total in-hospital systemic steroid dose and duration) was considered a post-treatment variable and therefore not used in propensity score estimation; it was summarised descriptively and explored in secondary analyses. Outcome measures were PTA gain (baseline minus discharge PTA), complete recovery (PTA gain ≥30 dB) and effective improvement (PTA gain ≥15 dB). Treatment intensity variables are summarised in Table 1.

**Table 1:** Treatment intensity and length of inpatient treatment in the full analytic cohort.

Variable	Systemic therapy (*n* = 240)	Intratympanic therapy (*n* = 44)	*p*-value
Total systemic steroid dose (mg)	231.5 ± 122.0	Not applicable	—
Duration of systemic steroids (days)	6.1 ± 2.8	Not applicable	—
Length of inpatient treatment (days)	9.8 ± 3.0	10.7 ± 2.7	0.041

Treatment intensity measures in the full analytic cohort. Total systemic corticosteroid dose and duration are reported for the systemic group; the intratympanic group had no systemic steroid exposure (not applicable). Because dose and duration are post-treatment variables, they were not included in propensity score estimation.

### Causal inference framework

Observational comparisons are vulnerable to confounding because patients who receive systemic or intratympanic therapy often differ in prognostic factors. Two broad statistical strategies exist to address this challenge: (i) modelling the treatment assignment and (ii) modelling the outcome. The treatment model estimates each patient’s probability of receiving systemic therapy conditional on baseline covariates (i.e., the propensity score) and can be used for matching, weighting or stratification to create comparable groups. The outcome model regresses the outcome on treatment and covariates to estimate the effect after adjustment. Each approach relies on correct specification of its respective model; if the propensity model is misspecified, matching may not fully remove confounding, whereas if the outcome model is misspecified, regression adjustment may yield biased estimates. The doubly robust (DR) estimator combines both models, providing two opportunities to control for confounding: unbiased estimates are obtained if either the propensity score model or the outcome model is correctly specified. This framework motivated our matching + regression approach.

### Propensity score matching

To control for measurable confounding we estimated the propensity score (PS)—the probability that each patient would receive systemic therapy—using a logistic regression model with only baseline covariates: age, sex, tinnitus, ear fullness, vertigo, hypertension, coronary heart disease, diabetes, baseline PTA, and time from symptom onset to treatment initiation. Propensity scores provide a summary measure of prognostic factors and can be used to balance treatment groups through matching, weighting or stratification (12–14). These baseline variables were chosen based on clinical judgment and prior literature as factors likely to influence both treatment choice and hearing prognosis. Treatment intensity variables were deliberately excluded because they are determined after the initiation of therapy and their inclusion can bias estimates (2, 3). For the matched sensitivity analysis, patients receiving systemic and intratympanic therapy were matched 1:1 using nearest-neighbour matching without replacement and a caliper width of 0.2 times the standard deviation of the logit of the PS. Common support was checked graphically. Balance between groups was assessed using the absolute standardised mean difference (SMD), with values <0.10 indicating acceptable balance (15). Balance diagnostics were summarised using a Love plot of absolute SMDs before and after matching. Unmatched patients were excluded from the matched analysis but remained in the weighting-based primary analyses described below.

### Doubly robust estimators (AIPW and matching + regression)

Primary analysis used augmented inverse probability of treatment weighting (AIPW) to estimate the average treatment effect (ATE) of initial systemic versus intratympanic corticosteroids in the full cohort. We estimated propensity scores as described above and combined inverse probability of treatment weighting with outcome regression to obtain doubly robust AIPW estimates (10, 11). For continuous outcomes we used linear regression and for binary outcomes logistic regression in the outcome model. To reduce sensitivity to extreme propensity scores we truncated estimated PS values at 0.01 and 0.99. Ninety-five percent confidence intervals for AIPW estimates were obtained using non-parametric bootstrap with 500 resamples. As complementary analyses we additionally reported stabilised IPTW and overlap-weighted estimates to assess robustness to limited common support (13, 16).

Because some baseline covariates remained imbalanced after matching (male sex, tinnitus, hypertension and diabetes had post-match |SMD| > 0.10), we implemented a doubly robust (DR) matched analysis. In the matched cohort we fitted multivariable regression models for each outcome with treatment group and the residual imbalanced covariates, and also included time to treatment to improve precision. For the continuous outcome (PTA gain) we used linear regression; for the binary outcomes (complete recovery and effective improvement) we used logistic regression. The linear model took the form.

Where (β1) is the adjusted mean difference between systemic and intratympanic therapy and (ε_i) is an error term. For binary outcomes we used logit models of the form.

Where (β1) is the log odds ratio for systemic versus intratympanic therapy. In the matched DR analysis, each model included the treatment indicator, residual imbalanced baseline covariates (male sex, tinnitus, hypertension, diabetes) and time to treatment. This “matching + regression” approach is doubly robust because it combines outcome regression with propensity score adjustment; an unbiased effect estimate is obtained if either the PS model or the outcome model is correctly specified (10, 11).

Across analyses, effect estimates are reported as mean differences (MD) in PTA gain and, for binary outcomes, both odds ratios (OR) and absolute risk differences (RD) to aid clinical interpretation. AIPW confidence intervals were obtained by non-parametric bootstrap (500 resamples), whereas regression and weighting models used robust standard errors. We also conducted a quantitative bias analysis using E-values for key binary outcomes to assess sensitivity to unmeasured confounding (17). All analyses were conducted in Python (version 3.11) using scikit-learn and statsmodels.

### Secondary analyses

Within the systemic therapy group we compared oral versus intravenous administration and intermediate- versus long-acting corticosteroids. Propensity score matching and doubly robust analyses were conducted analogous to the primary analysis. Because treatment intensity is similar across systemic subgroups, PS models included baseline covariates only. The corresponding subgroup analyses are provided in the Supplementary material for brevity.

## Results

### Patient selection and analytic samples

**Figure 1:**
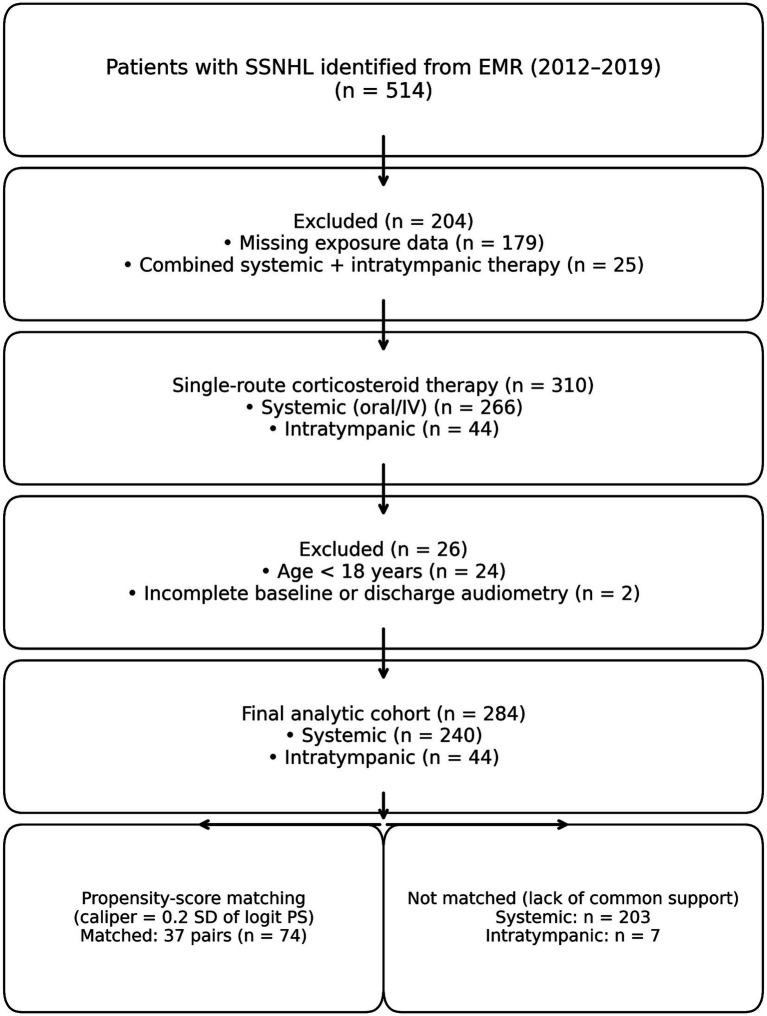
Participant flow diagram. Participant flow diagram showing inclusion/exclusion and analytic samples for weighting-based analyses (AIPW/IPTW/overlap weighting; full cohort) and propensity-score matching (matched subset).

Among 514 patients screened, 179 were excluded because the initial steroid route could not be reliably determined from the medical record and 25 were excluded because they received combined systemic and intratympanic corticosteroids, leaving 310 patients treated with a single corticosteroid route (systemic n = 266; intratympanic n = 44). We further excluded 24 patients aged <18 years and 2 patients with incomplete baseline or discharge audiometry, yielding a final analytic cohort of 284 adults (systemic *n* = 240; intratympanic *n* = 44). Figure 1 summarises the selection process.

For the matched sensitivity analysis, 1:1 propensity-score matching (caliper 0.2 SD of the logit PS) produced 37 matched pairs (*n* = 74). The remaining 203 systemic-treated patients and 7 intratympanic-treated patients were not matched because of limited common support and were excluded only from matched analyses. In contrast, the primary weighting-based analyses (AIPW, IPTW and overlap weighting) retained all 284 eligible adults.

### Baseline characteristics and balance diagnostics

**Figure 2:**
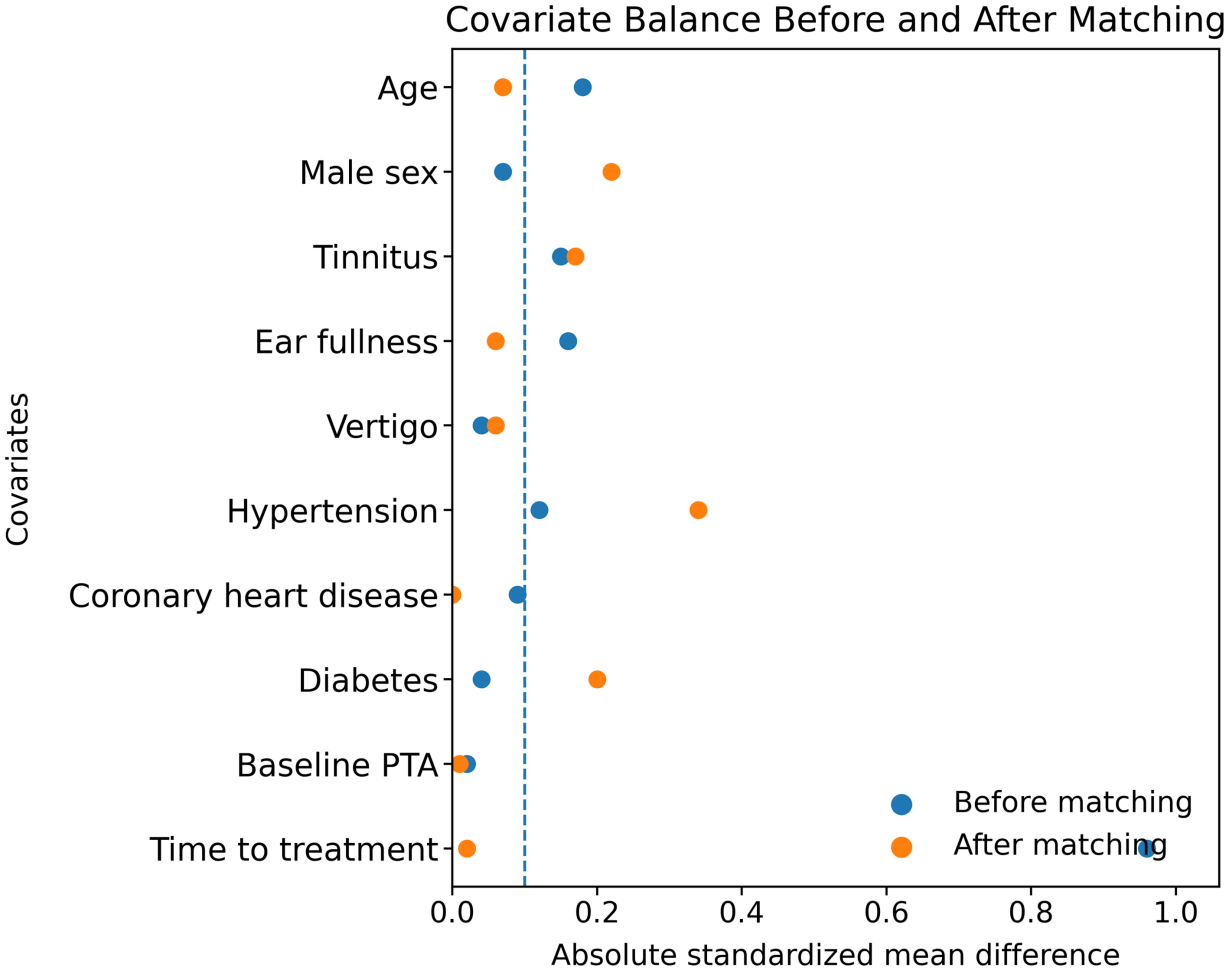
Love plot of standardized mean differences before and after matching. Love plot showing absolute standardized mean differences (SMDs) for baseline covariates before and after 1:1 propensity-score matching (caliper 0.2 SD of the logit PS). The dashed vertical line indicates |SMD| = 0.10. After matching, residual imbalance persisted primarily for male sex, tinnitus, hypertension, and diabetes; these covariates, together with time to treatment, were adjusted for in the matched doubly robust outcome models.

Baseline characteristics before and after matching are shown in Table 2. Prior to adjustment, patients receiving intratympanic therapy presented later (median time to treatment 14.5 vs. 5.0 days) and had a higher prevalence of vascular comorbidities (hypertension and diabetes), whereas baseline PTA was similar between groups. After matching on baseline covariates including time to treatment, covariate balance improved overall (Figure 2). Residual imbalance remained for male sex, tinnitus, hypertension and diabetes (post-match |SMD| > 0.10); these variables (and time to treatment) were therefore included in the matched doubly robust outcome models.

**Table 2:** Baseline characteristics before and after propensity-score matching.

Variable	Systemic therapy (*n* = 240)	Intratympanic therapy (*n* = 44)	Pre-match SMD	Systemic therapy (*n* = 37)	Intratympanic therapy (*n* = 37)	Post-match SMD
Age (years)	46.0 ± 14.1	48.5 ± 12.9	0.18	48.4 ± 15.1	47.5 ± 13.5	0.07
Male sex	117 (48.8%)	20 (45.5%)	0.07	14 (37.8%)	18 (48.6%)	0.22
Tinnitus	200 (83.3%)	39 (88.6%)	0.15	34 (91.9%)	32 (86.5%)	0.17
Ear fullness	71 (29.6%)	10 (22.7%)	0.16	11 (29.7%)	10 (27.0%)	0.06
Vertigo	93 (38.8%)	18 (40.9%)	0.04	11 (29.7%)	12 (32.4%)	0.06
Hypertension	33 (13.8%)	8 (18.2%)	0.12	10 (27.0%)	5 (13.5%)	0.34
Coronary heart disease	7 (2.9%)	2 (4.5%)	0.09	2 (5.4%)	2 (5.4%)	0.00
Diabetes	14 (5.8%)	3 (6.8%)	0.04	4 (10.8%)	2 (5.4%)	0.20
Baseline PTA (dB)	65.6 ± 22.7	65.0 ± 20.7	0.02	64.3 ± 21.2	64.1 ± 21.0	0.01
Time to treatment (days)	5.0 [3.0, 8.0]	14.5 [7.0, 30.0]	0.96	14.0 [7.0, 20.0]	13.0 [6.0, 30.0]	0.02

Data are presented as mean ± SD for continuous variables or number (percentage) for categorical variables. SMD, standardized mean difference.

### Primary comparison: systemic versus intratympanic therapy

In the crude cohort, mean PTA gain was 13.8 ± 14.8 dB with systemic therapy and 3.8 ± 18.9 dB with intratympanic therapy (MD 10.0 dB; 95% CI 5.0–14.9). In the primary AIPW analysis, systemic therapy remained associated with greater PTA gain (MD 10.1 dB; 95% bootstrap CI 1.8–19.5). Estimates were similar in stabilised IPTW (MD 10.5 dB; 95% CI 3.5–17.5) and were attenuated but directionally consistent under overlap weighting (MD 7.1 dB; 95% CI 2.0–12.2) and the matched doubly robust analysis (MD 5.6 dB; 95% CI − 1.6 to 12.8) (Table 3).

**Table 3:** Effect estimates for systemic versus intratympanic therapy under different analytic methods.

Analysis method	PTA gain (MD, 95% CI)	Complete recovery (OR, 95% CI)	Effective improvement (OR, 95% CI)
Crude (unadjusted)	9.95 (5.00, 14.89)	11.60 (1.56, 86.30)	1.77 (0.85, 3.67)
Stabilised IPTW	10.48 (3.49, 17.47)	13.44 (1.36, 132.53)	1.80 (0.84, 3.86)
Overlap weighting	7.14 (2.03, 12.24)	7.46 (0.67, 83.63)	1.38 (0.46, 4.13)
AIPW (primary)	10.10 (1.79, 19.54)	—	—
PSM + DR (matched)	5.60 (−1.58, 12.79)	3.13 (0.16, 62.19)	1.36 (0.36, 5.09)

Effect estimates for systemic versus intratympanic therapy under different analytic approaches. MD indicates the mean difference in PTA gain (dB). OR indicates odds ratio. Absolute risk differences (RDs) are reported in the text based on AIPW. The matched doubly robust models adjust for residual imbalances (male sex, tinnitus, hypertension, diabetes) and time to treatment. AIPW confidence intervals were obtained via bootstrap; regression and weighting models used robust standard errors.

For complete recovery, crude proportions were 21.3% in the systemic group versus 2.3% in the intratympanic group (OR 11.6; 95% CI 1.6–86.3). In the AIPW analysis, the estimated absolute risks were 20.0% versus 2.8%, corresponding to an absolute risk difference of 16.9% (95% bootstrap CI 9.4–23.4; number needed to treat ≈6). Stabilised IPTW yielded an OR of 13.4 (95% CI 1.36–132.5) with a similar absolute risk difference.

For effective improvement, crude proportions were 43.8% versus 31.8% (OR 1.77; 95% CI 0.85–3.67). The AIPW absolute risk difference was 11.4% (95% bootstrap CI − 15.3 to 30.9), indicating greater uncertainty for this endpoint. Overall, weighting and matched analyses produced directionally consistent estimates, but odds ratios had wide confidence intervals reflecting the small intratympanic sample size and low event counts (Table 3).

### Secondary comparisons

Treatment intensity differed by design between groups: systemic patients received systemic corticosteroids with a mean cumulative dose of 231.5 ± 122.0 mg over 6.1 ± 2.8 days, whereas intratympanic patients received local injections without systemic steroid exposure. Because total dose and duration occur after the initial route is selected, they were not included in the propensity score model; our primary estimand is therefore the effectiveness of the initial treatment strategy as practiced in routine care.

In an exploratory analysis restricted to systemic-treated patients, higher versus lower cumulative systemic dose (above vs. below the median) was not clearly associated with greater PTA gain [adjusted MD 1.25 (−3.42, 5.92)] or higher recovery rates (complete recovery adjusted OR 1.57 (0.78, 3.14); effective improvement adjusted OR 1.33 (0.74, 2.39)) after covariate adjustment. These subgroup analyses were underpowered and should be interpreted cautiously. Supplementary Figures 1–3 present matched-sample outcome distributions for transparency.

## Discussion

### Principal findings

In this retrospective single-center cohort of 284 adults with idiopathic SSNHL, we used a prespecified causal inference framework and doubly robust estimators to compare initial systemic versus intratympanic corticosteroid strategies. In the primary AIPW analysis adjusting for baseline covariates (including time to treatment), systemic therapy was associated with approximately 10 dB greater PTA gain and an absolute ~17% higher probability of complete recovery compared with intratympanic therapy. Evidence for effective improvement (≥15 dB gain) was less precise, with confidence intervals including no difference. Across stabilised IPTW, overlap weighting and matched doubly robust analyses, the direction of effect was consistent, although matched estimates were less precise due to limited covariate overlap.

### Methodological implications

A central challenge in observational SSNHL research is preserving correct temporal ordering in adjustment. Variables such as total steroid dose and duration are determined after selecting the treatment route and may lie on the causal pathway; adjusting for them can create post-treatment (over-adjustment or collider) bias (2, 3). We therefore restricted propensity score estimation to baseline covariates and treated treatment-intensity measures as post-treatment variables, summarising them descriptively and exploring within-route dose variation only as an exploratory secondary analysis. Importantly, we incorporated time from symptom onset to treatment initiation into both propensity score estimation and outcome models because delayed treatment is a well-recognised prognostic factor in SSNHL and could confound comparisons if unevenly distributed between groups (9).

Methodologically, our primary AIPW estimator combines inverse probability weighting with outcome regression and is “doubly robust,” remaining consistent if either the propensity score model or the outcome model is correctly specified (10, 11). Compared with relying solely on matching, weighting-based estimators improved statistical efficiency and reduced the impact of sample attrition. We also quantified sensitivity to unmeasured confounding using E-values: for complete recovery, the stabilised IPTW OR corresponded to an E-value of 26.4 for the point estimate and 2.1 for the lower confidence limit, suggesting that only a moderately strong unmeasured confounder would be required to move the confidence interval to include the null (17).

### Comparison with previous studies

Our findings should be interpreted alongside the broader evidence base. In a landmark randomised trial, Rauch et al. reported that intratympanic dexamethasone was non-inferior to oral prednisone for hearing outcomes in selected idiopathic SSNHL patients treated within 14 days (4). Systematic reviews and meta-analyses of first-line therapy have generally suggested comparable hearing outcomes between intratympanic and systemic steroids, but certainty is often low and conclusions vary due to heterogeneity in study design, dosing protocols, timing, and patient selection (5–8). In routine practice, intratympanic therapy is often reserved for patients who present later, have comorbidities, or have contraindications to systemic steroids, which can induce strong confounding by indication. By explicitly adjusting for baseline prognostic factors—including treatment delay—and applying weighting-based doubly robust estimators, our study provides complementary real-world evidence suggesting that initial systemic therapy may offer a clinically meaningful advantage in short-term hearing recovery and complete recovery rates in settings where systemic corticosteroids are feasible.

### Strengths and limitations

This study has several strengths, including an explicit causal inference framework, careful avoidance of post-treatment adjustment, transparent reporting of covariate balance, and the use of doubly robust estimators (AIPW and matched DR) with sensitivity analyses (stabilised IPTW and overlap weighting). Nonetheless, limitations warrant emphasis. First, residual confounding is possible because key prognostic factors were not available or incompletely recorded (e.g., smoking, viral prodrome, vascular risk burden beyond recorded diagnoses, audiogram configuration, imaging findings). Second, the intratympanic group was small and complete recovery events were rare, yielding wide confidence intervals for odds ratios and limiting precision. Third, treatment protocols were not standardised, and the estimated effect reflects the overall effectiveness of the initial treatment strategy as delivered in routine care, including any route-specific differences in dosing and follow-up. Fourth, outcomes were assessed using short-term in-hospital audiometry; longer-term hearing trajectories and patient-reported outcomes were not captured. Finally, the single-center design may limit external validity.

### Sample attrition and generalisability

Propensity-score matching retained 37 pairs (*n* = 74) from 284 eligible adults, meaning that approximately 74% of patients were excluded from the matched analysis because suitable matches were unavailable. Importantly, the matched cohort represents the region of covariate overlap—i.e., a subpopulation in which either initial strategy could plausibly have been selected (clinical equipoise). This strengthens internal validity by improving comparability, but it can reduce external validity because the matched estimand may not generalize to patients with more extreme prognostic profiles who were unmatched. In contrast, our weighting-based analyses (AIPW, IPTW and overlap weighting) retained all eligible patients and produced more precise estimates, improving generalisability to the underlying treated population while still relying on the no-unmeasured-confounding assumption.

### Implications and future research

Clinically, our results support systemic corticosteroids as a pragmatic first-line treatment strategy for idiopathic SSNHL when there are no contraindications, consistent with guideline recommendations to offer steroids early (9). Clinicians should counsel patients on potential systemic adverse effects and monitor comorbidities such as diabetes and hypertension (e.g., blood glucose monitoring and adjustment of hypoglycaemic therapy as needed). Intratympanic injections remain valuable for patients with contraindications to systemic steroids or who prefer to avoid systemic exposure, and as salvage therapy for incomplete recovery.

Future research should prioritise prospective, multi-center studies with standardised treatment protocols (route, dose, and timing), long-term hearing and quality-of-life outcomes, and richer capture of baseline risk factors. Advanced causal inference approaches—such as targeted maximum likelihood estimation or machine-learning-based propensity and outcome models—may further reduce bias and improve precision when applied with careful attention to temporal ordering and transparency.

## Conclusion

In this real-world retrospective cohort of adults with idiopathic SSNHL, initial systemic corticosteroids were associated with greater short-term PTA gain and a higher probability of complete recovery compared with intratympanic therapy. Weighting-based doubly robust estimators that retained all eligible patients produced directionally consistent estimates, while matched analyses were less precise due to limited overlap. These findings support systemic corticosteroids as a reasonable first-line strategy when not contraindicated and underscore the importance of rigorous causal inference methods in observational otology research.

## Data Availability

The datasets presented in this article are not publicly available because they contain patient information derived from electronic medical records and are subject to privacy and ethical restrictions. Requests to access the datasets should be directed to the corresponding authors.
