# Supplementary material for: Comparative effectiveness of initial systemic versus intratympanic corticosteroid therapy for sudden sensorineural hearing loss: a retrospective cohort study with doubly robust analysis
Source: Front Neurol. 2026 Apr 15;17:1752868. doi: 10.3389/fneur.2026.1752868 (PMC13124473; doi:10.3389/fneur.2026.1752868)

Supplementary Figures

Comparative effectiveness of initial systemic versus intratympanic corticosteroid therapy for sudden sensorineural hearing loss

Supplementary Figure 1. PTA gain after matching for systemic versus intratympanic therapy

Distribution of PTA gain (dB) in the propensity-score-matched sample (systemic vs intratympanic).

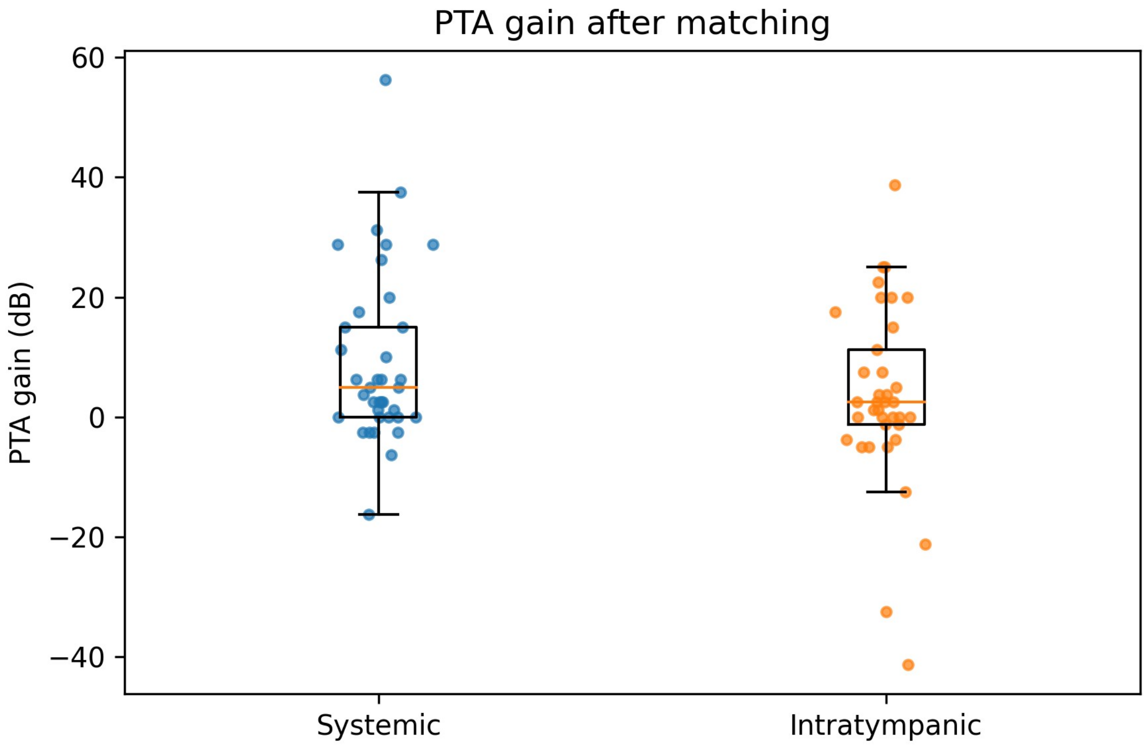

Supplementary Figure 2. Complete recovery after matching for systemic versus intratympanic therapy

Bars indicate proportions achieving complete recovery (≥30 dB gain) in the matched sample; error bars show 95% Wilson confidence intervals.

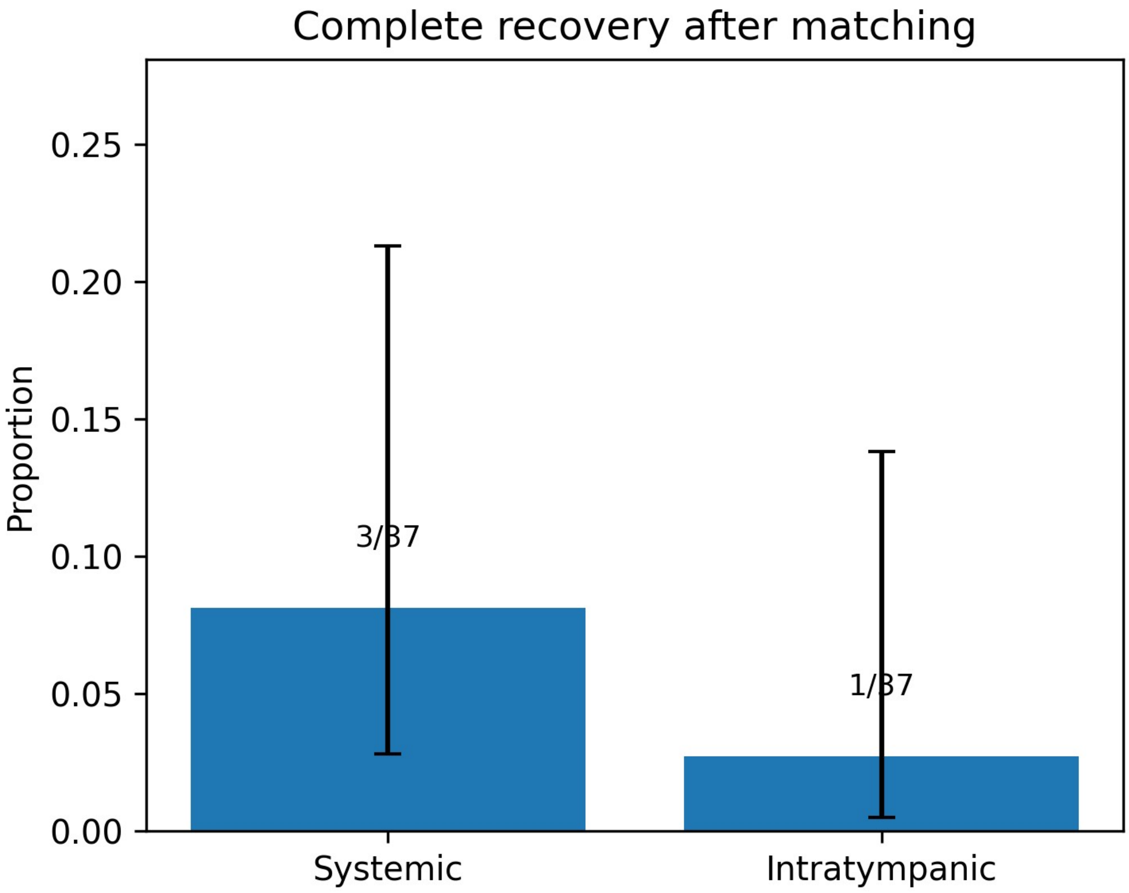

Supplementary Figure 3. Effective improvement after matching for systemic versus intratympanic therapy

Bars indicate proportions achieving effective improvement (≥15 dB gain) in the matched sample; error bars show 95% Wilson confidence intervals.

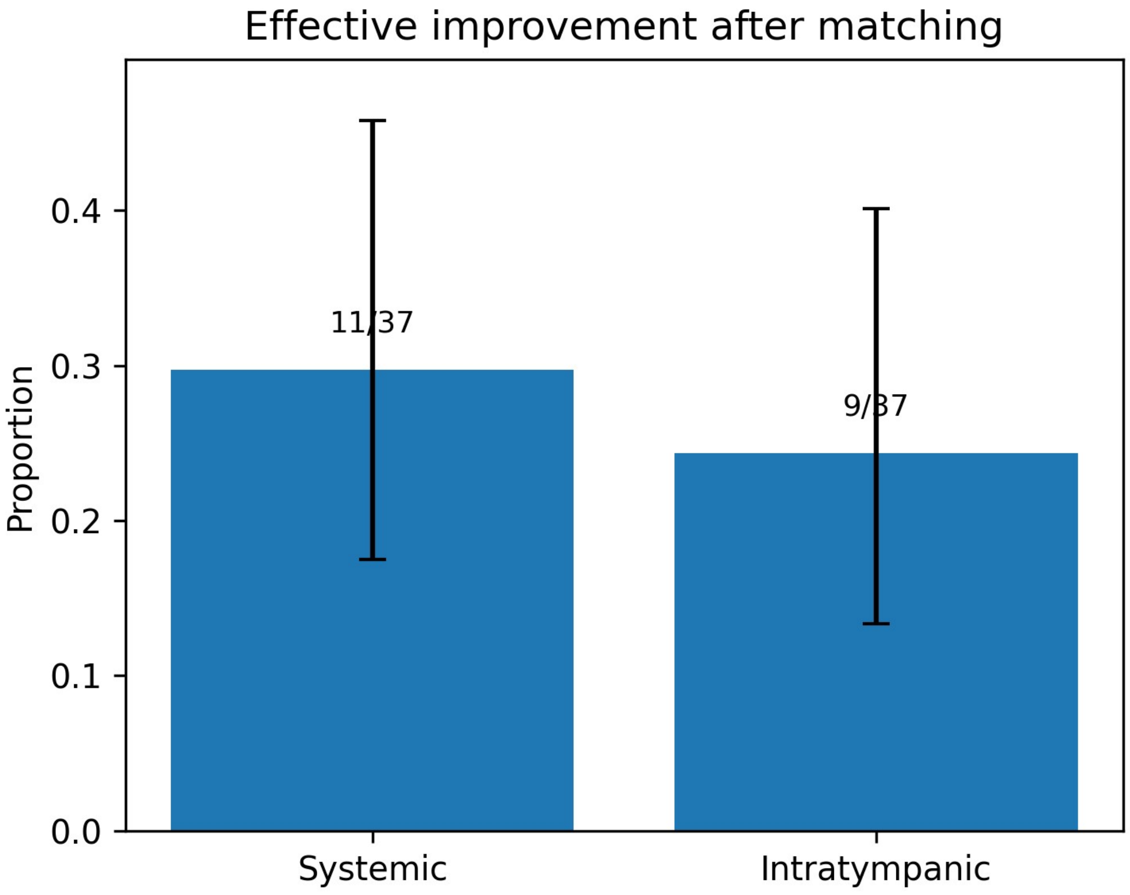

Supplement: Supplementary file 1 [file Data_Sheet_1.PDF]
